# Paper on Cerebro-Spinal Meningitis

**Published:** 1864-07

**Authors:** R. E. McVey

**Affiliations:** Waverly, Ills.


### PAPER ON CEREBRO-SPINAL MENINGITIS.

Read Befofe the Illinois State Medical Society at its Annual Meeting in May, 1864.

By R. E. McVEY, M. D., Waverly, Ills.

*Mr. President and Gentlemen :—*

The subject of cerebro-spinal meningitis has engaged the attention of the Profession for the last fifteen months. Certain portions of our country have suffered from an epidemic bearing the name referred to above. However, I think the name not very appropriate, as some of the patients die before inflammation could take place. Inflammation may follow as a secondary symptom, but the most prominent features of this disease at the commencement, are those of congestion and depression indicating the presence of some toxic agent in the circulating fluid, and that agent seems to have a very strong affinity for the brain, destroying the blood, and overcoming the contractile power of the capillaries and vessels of the brain. Consequently the circulation in the brain is interrupted, the capillaries and the coats of the vessels in the brain expand to accommodate the accumulation of fluid in their structure, which is evidenced by the epistaxis that follow in some of the cases that recover. I have seen patients die within six hours after they were taken with coma and prostration; the circulation giving out first, and intelection next,and lastly respiration, without any febrile reaction whatever, but in the majority of the cases reaction does take place in about six hours after the attack, and when reaction does take place, there is intense febrile excitement, with great restlessness and moaning. The eyes are suffused and the pupils are dilated. The patient is troubled with delirium, when aroused, for the most part answers questions correctly. Sometimes there are tremors of the whole body, one passing in succession after another, but more frequently but one side is affected. Sometimes there is squinting of one eye; the countenance has a brown hue; the tongue is slightly coated at the start, but as the disease progresses it becomes thicker; the bowels are constipated; the patient vomits a dark, grumous substance; the posterior cervical muscles contract, and draw the head backwards. Sometimes there is petechiæ over the neck, breast and extremities.

In regard to treatment, the most successful I have met with has been cerebral and arterial stimulants, with arsenic as an antiperiodic. It is of the utmost importance to arouse the capillaries and vessels of the brain, in order that they may relieve themselves of the fluid accumulated in their structures, and I know of no better agent than opium to do it with. Under the foregoing treatment I have had six patients recover and three die, and my partner, Dr. Brown, says he has treated, in the last six months, 21 cases, five of which were fatal, and sixteen recovered.

If the symptoms are severe, viz., coma, muscular contraction, with pain in the limbs, back and head, I have given opium in large doses, say, to an adult from 4 to 5 grs. Alcohol freely; usually Fowler's solution, from 6 to 8 gtts. every four hours, and when the muscular contraction continued for a considerable length of time, strychnia. Alteratives moderately, keeping the bowels open with mild aperients, invariably applying friction to the surface; tinct. capsicum and alcohol freely, with turpentine to the spine; sometimes blisters to the neck. Convalescence is generally slow.

Quinia I think, is contra-indicated, unless it is in very small doses, for the reason that it increases the agitation and produces a certain amount of deafness, with high delirium. Probably after the violence of the disease has passed off, it may be given with impunity, but not until the tremors have subsided and the brain so relieved that there is no danger of producing congestion of the eyes and ears. After this time it may have a salutary effect upon the digestive organs, and by giving tenacity to the system in general.

